# Possible Role of Human Herpesvirus 6 as a Trigger of Autoimmune Disease

**DOI:** 10.1155/2013/867389

**Published:** 2013-10-24

**Authors:** Francesco Broccolo, Lisa Fusetti, Luca Ceccherini-Nelli

**Affiliations:** ^1^Laboratory of Molecular Microbiology and Virology, Department of Public Health, University of Milano-Bicocca, Via Cadore 48, 20900 Monza, Italy; ^2^Dipartimento di Ricerca Traslazionale e delle Nuove Tecnologie in Medicina e Chirurgia, Centro Retrovirus, U.O. Complessa di Virologia Universitaria, Università degli Studi di Pisa, 56126 Pisa, Italy

## Abstract

Human herpesvirus 6 (HHV-6) infection is common and has a worldwide distribution. Recently, HHV-6A and HHV-6B have been reclassified into two distinct species based on different biological features (genetic, antigenic, and cell tropism) and disease associations. A role for HHV-6A/B has been proposed in several autoimmune disorders (AD), including multiple sclerosis (MS), autoimmune connective tissue diseases, and Hashimoto's thyroiditis. The focus of this review is to discuss the above-mentioned AD associated with HHV-6 and the mechanisms proposed for HHV-6A/B-induced autoimmunity. HHV-6A/B could trigger autoimmunity by exposing high amounts of normally sequestered cell antigens, through lysis of infected cells. Another potential trigger is represented by molecular mimicry, with the synthesis of viral proteins that resemble cellular molecules, as a mechanism of immune escape. The virus could also induce aberrant expression of histocompatibility molecules thereby promoting the presentation of autoantigens. CD46-HHV-6A/B interaction is a new attractive mechanism proposed: HHV-6A/B (especially HHV-6A) could participate in neuroinflammation in the context of MS by promoting inflammatory processes through CD46 binding. Although HHV-6A/B has the ability to trigger all the above-mentioned mechanisms, more studies are required to fully elucidate the possible role of HHV-6A/B as a trigger of AD.

## 1. Introduction

Human herpesvirus 6 (HHV-6) infection is common and has a worldwide distribution. Recently, HHV-6A and HHV-6B have been reclassified into two distinct species based on different biological features (genetic, antigenic, and cell tropism) and disease associations: HHV-6A, with still unknown disease association, and HHV-6B, the etiologic agent of roseola (*exanthem subitum*), a childhood benign febrile disease.

In the recent years, several reports have provided important information linking HHV-6A/B to autoimmune diseases (AD) including multiple sclerosis [1–7], autoimmune connective tissue diseases [8–11], and Hashimoto's thyroiditis [12]. In addition, it has been suggested that HHV-6A/B infection might be related to the onset of autoimmune disorders, including Sjogren's syndrome [13], purpura fulminans, severe autoimmune acquired protein S deficiency [14], severe and acute autoimmune hepatitis [15, 16], and autoimmune hemolytic anemia/neutropenia [17].

The focus of this review is to discuss the above-mentioned AD associated with HHV-6 and the mechanisms proposed for HHV-6A/B-induced autoimmunity. HHV-6A/B might trigger autoimmunity by exposing high amounts of normally sequestered cell antigens, through lysis of infected cells. The virus could also induce aberrant expression of histocompatibility molecules thereby promoting the presentation of autoantigens. Another potential trigger is represented by molecular mimicry, with the synthesis of viral proteins that resemble cellular molecules, as a mechanism of immune escape. Based on the similarity in peptide sequence between viral proteins and self-proteins, it has been postulated that viral infections could activate cross-reactive T cells, able to recognize both viral and self-antigens, which could then trigger an autoimmune response and cause tissue damage [18–20].

## 2. HHV-6 and Multiple Sclerosis

HHV-6A/B has long been cited as a potential candidate virus for the etiology of multiple sclerosis (MS), an inflammatory, demyelinating disease of the central nervous system, believed to be initiated and mediated by autoreactive T cells directed against myelin antigens. It develops in young adults with a complex genetic predisposition and is thought to require an inciting environmental insult such as a viral infection to trigger the disease [21]. HHV-6A infects and can establish latency in the central nervous system (CNS). HHV-6A and HHV-6B have specie-specific tropisms in human glial cells, suggesting that the two HHV-6 species might be responsible for distinct disease outcomes due to the infection pattern in these cells *in vivo* [22]. HHV-6A (which has been more often associated with MS) established a productive infection in astrocytes with cytopathic effect and high virus DNA loads, while the HHV-6B infected astrocytes showed no morphological changes but maintained low levels of intracellular viral DNA without detectable RNA. A relationship between HHV-6A and MS was first suggested by immunohistochemical demonstration of viral antigen in oligodendrocytes of MS white matter lesions but not in control brains [1]. Since this initial report, several studies have supported an association of HHV-6A/B and MS by the demonstration of elevated antibody titers to HHV-6A/B antigens compared to controls and by amplification of HHV-6A/B DNA from the serum, cerebrospinal fluid (CSF) and brain tissue of MS patients [1–7, 21–29]. Exacerbation of relapsing-remitting MS has been linked to higher viral loads in serum and in peripheral blood mononuclear cells (PBMCs), suggesting association of HHV-6A/B reactivation with disease relapses [2, 26, 30]. Abundant clinical studies have highlighted a correlation between MS and several parameters assessing for HHV-6A/B infection. For instance, the levels of HHV-6A/B DNA in the serum, which are characteristic of ongoing infection, are significantly increased in MS patients when compared to healthy donors or with patients with other diseases [1]. HHV-6DNA was also detected at higher frequencies in the CSF and in the peripheral blood mononuclear cells of MS patients [2, 6, 26]. Moreover, the levels of HHV-6A/B-specific IgG and IgM in the serum and in the CSF were reported to be higher in MS patients in several studies [2], although this phenomenon does not appear to be specific for HHV-6. Soldan et al. also showed that lymphoproliferative responses against HHV-6 antigens were increased in MS patients [2]. The analysis of brain biopsies and postmortem tissues indicated that HHV-6A/B DNA was present more frequently in the brain of MS patients than in control brains and that it was also more frequent in MS lesions than in normal areas of the same brains. Immunohistochemistry analyses confirmed the presence of viral proteins in oligodendrocytes and astrocytes in the brain from MS patients, with a higher frequency in demyelinating plaques [3, 24, 29, 31]. Most interestingly, viral loads were detected more frequently, and levels of HHV-6A/B-specific IgG were increased in MS patients experiencing disease exacerbation [21, 31, 32], thus suggesting a correlation between HHV-6A/B infection and MS relapses. As the distinction of HHV-6A and -6B as two distinct viruses was only recently adopted, many of the initial studies do not discriminate between the two species. However, based on few reports, it appears that HHV-6A is found more frequently than HHV-6B in the serum of MS patients [23]. Especially in case of active infection, Álvarez-Lafuente et al. have found only HHV-6A [31]. In contrast, in one study, intrathecal HHV-6B IgG levels were more abundant than HHV-6A IgG in MS patients, and only HHV-6B-specific IgM levels were found [32]. The potential association between HHV-6A and HHV-6B infection and MS has often been discussed and remains controversial. Some studies provided contradictory results [33, 34], raising methodological and technical questions, especially concerning the choice of control groups and the immunological state of the included patients, who often receive immunosuppressive treatments that may provoke latent herpesvirus reactivation by itself. 

### 2.1. Pathogenic Hypotheses for HHV-6A-Induced MS

New studies investigating the biology of HHV-6A have given insights towards understanding how HHV-6A may play a role in MS pathology. By inducing molecular mimicry or excessive complement activation, HHV-6 reactivation may have the potential to trigger autoimmunity and tissue damage associated with MS lesion development. Reports suggested that constitutive presence of active HHV-6A/B infection in glial cells in inflamed CNS tissue could result in virus-triggered immunopathologies in MS [31]. A mechanism of molecular mimicry involving HHV-6A has been proposed as one mechanism by which the autoimmune process could be triggered. One study showed that certain residues on the HHV-6A genome are identical to residues of myelin basic protein. Importantly, both T-cells and antibody responses to this peptide sequences were found elevated in MS patients [35]. Moreover, *in vitro* infection of glial precursor cells was found to impair cell replication and increase the expression of oligodendrocyte markers, suggesting that HHV-6A infection of the CNS may influence the neural repair mechanism; lymphoproliferative response to HHV-6A antigens has been also demonstrated to be greater in MS patients than in controls [36]. Yet, whether HHV-6A infection is the etiologic cause, a factor for disease progression, or a consequence of MS remains unclear and would need further investigation. Taken together, epidemiological data and the presence of active HHV-6A infection in some MS brain samples suggest a possible role for HHV-6A in perpetuating tissue damage in MS. 

Several studies suggest that such a mechanism could be involved in HHV-6A-induced neuroinflammation. A first study reported that 15%–25% of HHV-6-specific T cell clones obtained from healthy donors or MS patients were cross-reactive to myelin basic protein (MBP), one of the autoantigens implicated in MS pathology [37]. In fact, MBP and the U24 protein from HHV-6 were later shown to share an identical amino acid sequence of 7 residues. Moreover, T cells directed against an MBP peptide also recognized an HHV-6A peptide, both peptides containing the identical sequence. Interestingly, cross-reactive cells were more frequent in MS patients than in controls [35]. These data were further confirmed by a more recent study, in which the presence of cross-reactive CD8+ cytotoxic T cells was found [38]. Altogether, these studies suggest that HHV-6A infection can activate T cell responses, which can simultaneously be directed against myelin sheaths, thus strongly supporting the potential role for HHV-6A in autoimmune diseases affecting the CNS.

Moreover, the fact that HHV-6A/B is ubiquitous virus that infects the vast majority of humans pose a relevant question about how only a minority of individuals is affected by MS. In this regard, a complex interaction between these pathogens and the individual genetic background represents the most reasonable explanation. Indeed, besides the well known risk conferring genes belonging to the HLA DR locus [33], a recent paper claimed that some KIR genes are strongly underrepresented amongst MS patients with an increased risk of disease susceptibility amongst the carriers of the natural HLA-C ligands (HLA C1) [34]. KIRs are MHC class I-specific regulatory receptors utilized by human natural killer (NK) cells and CD8 T cells [35]. Several lines of evidence link differences in KIR expression to differential responses to invading pathogens and autoimmune disorders [36, 37]. Since the authors demonstrated that NK recognition and specific killing of HHV-6 infected lymphocytes are tightly regulated by KIRs and their MHC-ligands [38], it will be important to establish the role of KIRs in MS with a particular attention on HHV-6 viremic MS patients. 

Finally, CD46-HHV-6 interaction is a new attractive mechanism proposed: HHV-6 could participate in neuroinflammation in the context of MS by promoting inflammatory processes through CD46 binding. CD46 or membrane cofactor protein (MCP) is a member of the complement regulatory protein family, and it is also known to be a receptor for different viruses and bacteria. In 1999, CD46 was identified as the cellular receptor for HHV-6A entry [39]. Recently, Tang et al. [40] demonstrated that CD134, a member of the TNF receptor superfamily, functions as a specific entry receptor for HHV-6B. Although HHV-6B and -6A share 90% identity in their nucleic acid sequence, they show distinct pathogenesis and cell tropism. The discovery of an HHV-6B-specific receptor supports the idea that the use of different receptors by HHV-6A and -6B is an important biological feature underlying their different characteristics and disease manifestations.

Two forms of CD46 have been described: membrane and soluble (sCD46), but no conclusive evidence is documented in the literature to show whether posttranslational events or alternative splicing produces the soluble form [41], whose levels have been shown to be increased in the serum of patients with autoimmune disorders including Sjogren's syndrome [42], systemic lupus erythematosus [43], and MS [44]. Besides, a physical association between the HHV-6 virion and sCD46 was found in the serum of patients with MS with HHV-6 DNA, but not in the serum of controls, suggesting that the presence of HHV-6/CD46 complexes might contribute to the increased levels of CD46 found in the serum of patients with MS [45]. 

Regarding previous results on CD46, in a study performed by Hammarstedt et al. [46] with the objective of identifying possible host proteins associated with HHV-6, Western blot analyses showed that the cellular complement protein CD46 was associated with the purified and infectious virions; the authors suggested that the relevance of the association in disease and especially in autoimmunity will be further investigated. As it has been demonstrated that CD46 is selectively and progressively downregulated from the target cell surface during the course of HHV-6 infection [39], the increased levels of CD46 expression reported in this study in patients with MS with HHV-6 infection could be related to the increased levels of the soluble form of CD46 described in patients with MS [44, 45], and, therefore, this could constitute an immunopathogenic factor that should be investigated in MS. However, many theories could be exposing to explain the different ways in which CD46-HHV-6 interaction can play a role in MS pathogenesis. One of them is related to the persistence of the virus in the serum. In has been described that some patients with MS had HHV-6 genomes in their sera in consecutive samplings performed over 1-year followup [46]; this way, if HHV-6 is attached to the receptor in its soluble form, different epitopes of the virus that share homology with human proteins and also different host cell proteins that could be incorporated to the envelope of the virus [47] would be more exposed to the host immunological system, increasing their possibilities in playing a role in autoimmunity. Furthermore, CD46 is highly expressed at the blood-brain barrier (BBB) [48] that is composed of tight junctions, which prevent the entry of large proteins into the CNS; crossing this barrier is precisely regulated and crucial for the immune surveillance of the brain, but CD46 could be mediating access of HHV-6 to the brain by promoting passage of the BBB. However, as we have previously mentioned, CD46 is also a member of the complement regulatory protein family that confers protection against activated complement-mediated lysis by inactivating C3b/C4b deposited on the membrane of autologous cells; therefore, the increased levels of CD46 demonstrated in the serum and CSF of patients with MS may be indicative of an increased activation of the complement system in MS, both peripherally and intrathecally, and lend further support to the possible contribution of complement in disease pathogenesis. CD46 has also been related to T cells: it is known that CD3/CD46 costimulation promotes T-cell proliferation with a potency comparable with that of CD28 [49], and the importance of CD46 in the regulation of the immune response through the induction of Tr1 cells and IL-10 production has been highlighted [50]; as the role of regulatory T cells in patients with MS has been previously demonstrated by various groups [51, 52], an alteration in CD46 in patients with MS could lead to further damage and inflammation [50]. However, the real significance of CD3/CD46 costimulation *in vivo* remains unknown.

Finally, this complement regulatory protein also plays an important role in the adaptive immune response as it can modulate T cell responses depending on which cytoplasmic tail is expressed [53] and can induce CD4+ T cells toward a Tr1 phenotype, with high IL-10 production [54]. One could then hypothesize that HHV-6A/B, by binding to their receptor, could modulate its functions. In support to this theory, a clinical study indicated that increase in HHV-6A/B viral load was correlated to enhanced CD46 expression in MS patients [55], and several alterations in CD46 functions were described; the CD46-induced IL-10 secretion by T cells was strongly decreased [55], whereas the CD46-dependant IL-23 production by DC and IL-17 expression by T cells were enhanced [56, 57]. This suggests that HHV-6 could participate in neuroinflammation in the context of MS by promoting inflammatory processes through CD46 binding.

## 3. HHV-6 and Autoimmune Connective Tissue Diseases

Autoimmune connective tissue diseases (ACTD) encompass a very large group of diseases, including systemic sclerosis or scleroderma (SSc), systemic lupus erythematosus (SLE), discoid lupus erythematosus (DLE), dermatomyositis (DM), rheumatoid arthritis (RA), and other conditions causing chronic inflammation that can affect many organs and systems. Although the etiology of ACTD remains unclear, clinical, epidemiological, and laboratory findings suggest that several viral infections may be involved in these diseases [58]. Reactivation of HHV-6A/B, as suggested by the high rates of viral isolation, occurs frequently in patients with collagen vascular diseases [8]. Moreover, Hoffmann and coauthors demonstrated active HHV-6A/B infection in a 37-year-old woman affected by SLE and histiocytic necrotizing lymphadenitis (Kikuchi-Fujimoto disease) [9]. More recently, we detected frequent reactivation of HHV-6 in active ACTD (especially in lupus erythematosus) [10, 11]. Our data suggest that HHV-6A/B may act as a pathogenic factor predisposing patients to the development of ACTD or, conversely, that these disorders may predispose patients to HHV-6A/B reactivation [11].

### 3.1. Pathogenic Hypotheses for HHV-6A/B-Induced ACTD

A number of infectious agents including members of the Herpesviridae family and Parvovirus B-19 (B19V) have been proposed as possible triggering factors in ACTD, mainly in SSc [18–20, 58–60]. Homology between viruses and autoantibody targets suggests that molecular mimicry may play a role in the initiation of antibody response in disorders characterized by diffuse vascular damage. Four pathogenic hypotheses have been proposed: molecular mimicry, endothelial cell damage, super-antigen stimulation, and microchimerism [18–20, 58–62]. However, evidence for a direct association is still lacking, even though several studies have provided important information linking infectious agents to ACTD [62]. Indeed, infectious agents have the potential to initiate autoreactivity through polyclonal activation and the release of previously sequestered antigens or molecular mimicry. Evidence from animal models indicates that molecular mimicry of host proteins by a pathogen can induce autoimmune diseases [19], although it appears to be an infrequent occurrence in the majority of viral infections. More commonly, viruses induce autoimmunity by cell death, predominantly by increased apoptosis, resulting in the release of self-antigens; increased apoptosis indeed has been suggested as a major pathogenetic mechanism in ACTD [20]. 

## 4. HHV-6 in Hashimoto's Thyroiditis

Hashimoto's thyroiditis (HT), or chronic lymphocytic thyroiditis, is a common autoimmune disease with unknown etiology, and its prevalence has been increasing over the past 50 years [63, 64]. Together with genetic factors, environmental factors are thought to be important in triggering autoimmune thyroid diseases (AITD), and viral infections have been suggested as possible environmental triggers [65], yet no conclusive evidence is available. Also herpesviruses have been suggested as potential cofactors and have occasionally been detected in AITD [66, 67]. Thyroid cells infected with human cytomegalovirus were shown to act as antigen presenting cells and therefore might be involved in autoimmunity [68], patients with Graves' disease display a higher frequency of EBV-infected B cells secreting antibody to TSH-R [69], and AITD patients have elevated antibody titers against EBV antigens [70]. HHV-6A/B DNA has been detected in HT tissue specimens but not in tissues from Graves' disease or multinodular goiter [67]. More recently, a 2012 study [12] has linked HHV-6A to Hashimoto's thyroiditis (HT). The study found that HHV-6A was detected significantly more frequently among thyroid fine needle aspirates (FNA) from HT individuals than controls (82% versus 10%, resp.), and low-grade acute infection was identified in all HHV-6 positive HT samples compared to 0% of controls. Furthermore, the presence of HHV-6A infection was found localized mainly to thyrocytes, rather than in lymphocytes infiltrating the lesion, and increased prevalence of latent HHV-6A infection was seen in PBMCs overall. In addition, the group demonstrated that thyroid cells infected with both HHV-6A and HHV-6B became susceptible to NK-mediated killing, providing evidence of a potential mechanism for HHV-6A/B-induced autoimmunity. These findings are consistent with the possibility that the thyroid of HT patients may constitute a site of active HHV-6A/B infection/replication. 

### 4.1. Pathogenic Hypotheses for HHV-6-Induced HT

Recently, the Italian research group [12] provided evidence indicating that HHV-6A/B may induce a *de novo* expression of HLA class II molecules in thyrocytes, which may thus behave as functional antigen presenting cells for CD4+ T lymphocytes. Intriguingly, in this same study the enhanced HHV-6A/B-specific T cell responses were observed in all HT patients, with a marked increase in the number of CD4+ T lymphocytes recognizing HHV-6A/B antigens, particularly the subset of polyfunctional CD4+ T cells secreting both IFN-*γ* and IL-2. These findings are consistent with an abnormal, probably persistent, immune response to HHV-6A/B antigens in HT patients, possibly favored by the local upregulation of HLA class II molecules on thyrocytes induced by HHV-6A/B infection. However, further studies are required to fully elucidate this association and the mechanisms underlying the possible role of HHV-6A/B as a trigger of HT. 
